# What are the hidden shortcomings of balance training research in older adults that prevent its transfer into practice? Scoping review

**DOI:** 10.1371/journal.pone.0308752

**Published:** 2025-01-02

**Authors:** Zuzana Kováčiková, Iveta Cimboláková, Marcel Čurgali, Jana Labudová, Erika Zemková

**Affiliations:** 1 Department of Gymnastics, Dance, Fitness & Combat Sports, Faculty of Physical Education and Sport, Comenius University in Bratislava, Bratislava, Slovakia; 2 Institute of Physical Education and Sport, Pavol Jozef Šafárik University in Košice, Košice, Slovakia; 3 Department of Physical Education and Sport, Faculty of Education, Constantine the Philosopher University in Nitra, Nitra, Slovakia; 4 Department of Outdoor Sports and Swimming, Faculty of Physical Education and Sport, Comenius University Bratislava, Bratislava, Slovakia; 5 Department of Biological and Medical Sciences, Faculty of Physical Education and Sport, Comenius University in Bratislava, Bratislava, Slovakia; National center for chronic and non-communicable diesease prevention and control, CHINA

## Abstract

**Background:**

Although a lot of attention is paid to the flaws of balance training research in older adults, the low methodological quality and incomplete reporting of studies still limit the knowledge transfer between research and practice. These known shortcomings are considered also as barriers for creating recommendations for balance training in older adults. Despite the considerable efforts to improve the scientific quality of studies, such recommendations have not yet been formulated to date. Therefore, this scoping review aims (1) to analyze the literature that addresses balance training in older adults, (2) to identify and summarize gaps in the existing literature, and (3) to propose future research on this topic.

**Methods:**

We focused on studies that evaluated the effect of balance training on balance control in apparently healthy older adults over 60 years of age.

**Results:**

Out of 6910 potentially relevant studies, only 26 met the eligibility criteria. The identified shortcomings were as follows: missing a priori criteria for training session attendance and leisure-time physical activities, insufficiently described exercises and training load, and inappropriately chosen tests.

**Conclusions:**

Among the shortcomings of the balance training research, the insufficiently described balance training program and inappropriately chosen tests can be considered the most important. For this reason, even with an excellently designed experiment, it is almost impossible for practitioners to apply the results of such studies into practice. Therefore, researchers should pay more attention to possible users of the acquired knowledge, which is more than desirable in the case of exercise programs for older adults.

## Introduction

Even though balance training is highly recommended to older adults for improving balance control [1, 2], reducing the risk and occurrence of falls [3], improving physical functions [4] and independence in performing activities of daily living [5], consensually accepted training guidelines are still not available. We register individual efforts to propose some kind of universal recommendations for the balance training of older adults. These are usually limited to the duration of the intervention, training frequency and volume. Moreover, the recommendations are often contradictory. For instance, Lesinski et al. [6] found that effective balance training in relatively healthy older adults is characterized by training period of 11–12 weeks, with a frequency of 3 training sessions/week and a length of 31–45 min. However, the desired training effect can be achieved even in a shorter period, with a lower frequency of training units and training duration (6 weeks, 2 training sessions/week for 30 minutes) [7]. To determine which study is more believable, it is necessary to consider other aspects such as its methodological and reporting quality.

Reliable tools that facilitate a comprehensive review of exercise training trials such as TESTEX [8] or PEDro scale [9] have been proposed to address common shortcomings in study design, quality, and reporting in the intervention studies. However, none of the available tools include detailed evaluation of training protocol. This is perhaps due to the assumption that the training protocol as the cornerstone of intervention studies must be always well described.

Methodological quality of balance training studies is usually moderate to weak [6]. Within the training protocol, deficiencies are most often attributed to training modalities, such as the number of training exercises, dosage, and duration. Despite these known shortcomings of individual studies, guidelines for balance training in older adults have still not been formulated. Therefore, we assume that there are also other, and so far, undescribed shortcomings of balance training studies that can be considered as barriers for implementing evidence-based findings into clinical and training practice.

Therefore, the aim of this scoping review is to identify the shortcomings of balance training studies related primarily to the description of training protocol. We focused on studies conducted in apparently healthy older adults. The reason is that various comorbidities and disease severity may require different approaches to creating protocols, which is then reflected in the high methodological diversity between studies. The very high methodological diversity of studies is an obstacle to reaching a scientific consensus on recommendations regarding balance training for older adults [10].

## Methods

### Research question

What are the methodological gaps of balance training studies in apparently healthy older adults?

### Design

#### Scoping review

A scoping review can be used as a standalone project or as a preliminary step to a systematic review to identify studies shortcomings and research gaps on the specific topic in the existing scientific literature [11]. The methodological framework JBI Manual for Evidence Synthesis was used (Chapter 11) [12]. This review was reported according to the PRISMA Extension for Scoping Reviews reporting guidelines [13].

### Inclusion and exclusion criteria

A PCC framework was used to guide the eligibility criteria for this scoping review [14]. Inclusion and exclusion criteria are listed in Table 1.

**Table 1:** Eligibility criteria.

	Inclusion	Exclusion
**Population**	Older adults aged 60 years and over in apparently good state of health*.	People < 60 years.
		Missing information about age.
	*Apparently healthy older adults referred to people who were community dwelling; physically independent; without a history of serious neurological, cardiovascular, orthopedic, or other diseases affecting balance.	
		Presence of disease/s in a health anamnesis.
		Performance of normal daily activities with aids.
**Concept**	Studies aimed at evaluating the effect of balance training* on balance control.	Multimodal interventions.
	*Balance training was defined as a single session of balance exercise/task or sequence of training units using postural equilibrium exercises, aimed at improving balance control over a specific time.	
		Studies in which balance was not evaluated.
**Context**	Open	–
**Type of evidence sources**	Randomized controlled trials, non-randomized controlled trials, quasi-experimental, before and after studies, prospective and retrospective cohort studies, and case-control studies.	Conference papers, abstracts, theses, books, research reports, and protocols.
		To avoid duplication of literature, systematic review studies* were excluded.
		*Systematic review studies are usually considered secondary studies and are therefore not recommended for inclusion in a scoping review.

### Search strategy

**Fig 1:**
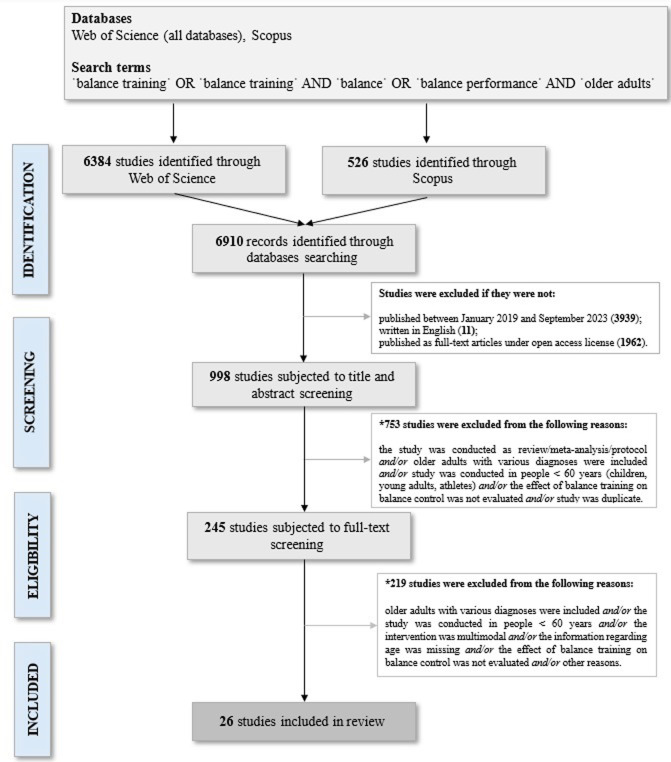
PRISMA flow diagram. * It was not possible to determine the exact number of excluded studies within each category. This was because some studies could be excluded for several reasons at the same time. For example, the effect of balance training was assessed in young adults with chronic disease.

A literature search was conducted through the electronic databases Web of Science (all databases) and Scopus. The search strategy included the following combination of terms: ’balance training’ OR ’balance intervention’ AND ’balance’ OR ’balance control’ AND ’older adults’. Only articles published in a peer-reviewed journals in the period January 2019—August 2023, full-text articles written in English, and articles published under an open access license were included. We focused on the recent articles to reflect more up-to-date shortcomings in the field of balance training research in older adults. See Fig 1 for a PRISMA flow diagram.

### Source of evidence selection

A pilot evaluation was conducted on 20 randomly selected articles dealing with balance training in older adults. Google Scholar was used to search for articles. Three reviewers analyzed them according to the pre-defined criteria within the protocol. The aim was to verify the consensus of all reviewers in the data coding. If there were disagreements in the coding between the reviews, areas of disagreement were discussed until 100% agreement was achieved between reviewers. Identified studies were exported to an MS Excel document in the form of study title, author(s), publication year and source.

### Data extraction

Study protocol included information about author(s) and year of publication; number of participants (including gender statistics); balance training type; training characteristics such as frequency of training units and duration; type of trained balance; training protocol description; additional physical activity; a priori criteria for session attendance; control group; diagnostic tools; and the outcome (Supplementary file 1).

## Results

A total of 6910 potentially relevant studies were identified based on the key terms. Of these, 26 studies met the eligibility criteria and were included in this scoping review. The most studies were published in 2021 (12 out of 26). All included studies are processed according to outcome categories in Table 2.

**Table 2:** Monitored outcome categories of balance training studies in apparently healthy older adults.

Study	Type of trained balance	BT description	APA performed along with intervention	APA restricted/prohibited within criteria	Type of control group	A priori criteria for session attendance	Tests
Aviles et al. [23]	Dynamic reactive	No	No	Yes	Control intervention	Yes	TUG
							OLS
							BBS
							POMA
							MSL
							Perturbed walking on a treadmill
Okubo et al. [3]	Dynamic reactive	Yes	Yes (not described)	No	Control intervention	No	3D Motion Analysis
Allin et al. [24]	Dynamic reactive	Yes	No/Not mentioned	No	Control intervention	No	3D Motion Analysis
							(perturbed walking)
							TUG
							OLS
							POMA
Anders et al. [25]	Dynamic proactive	Yes	NA*	NA*	None	NA	3D Motion Analysis (game)
Aviles et al. [26]	Dynamic reactive	Yes	No/Not mentioned	No	Control intervention	No	BBS
							Trunk kinematics during perturbed walking
Jeon et al. [27]	Dynamic proactive	No	No/Not mentioned	No	Control intervention	No	TUG
							POMA
							FRT
Granacher et al. [28]	Static	Yes	No/Not mentioned	No	Passive	No^#^	TUG
							FRT
							Romberg test
	Static reactive						Static posturography (EO/EC/foam)
Higgins et al. [29]	Static reactive	Yes	No/Not mentioned	No	Control intervention	No	Standing on the wobble board with an inertial measurement unit
Javadpour et al. [30]	Static	No	No/Not mentioned	No	Passive	No^#^	FAB scale
	Static proactive						
	Static reactive						
	Dynamic						TUG
	Dynamic reactive						
	Dynamic proactive						
Khushnood et al. [31]	Static proactive	No	No	Yes	Control intervention	No	TUG
	Dynamic proactive						
Khushnood et al. [32]	NA^¥^	No	No	Yes	Control intervention	No	TUG
							Fukuda Step Test
Martelli et al. [33]	Dynamic reactive	Yes	NA*	NA*	Control intervention	NA	3D Motion Analysis
							(perturbed walking)
Rezaei et al. [34]	Static	No	No/Not mentioned	No	Control intervention	No	FAB scale
							Biodex Balance System
	Static reactive						
							(bipedal stance)
Sadeghi et al. [35]	BT group	Yes	No/Not mentioned	No	Passive	Yes	OLS (foam/hard)
							Tandem stance
	Static						
	Static proactive						
	Dynamic proactive						
	VR group						TUG
							10MWT
	NA^¥^						
	BT +VR group						
	NA^¥^						
Sápi et al. [36]	NA^¥^	No	No/Not mentioned	No	None	No	m-CTSIB test on NeuroCom Balance Master
Song et al. [37]	Dynamic	Yes	NA*	NA*	Own controls	NA	3D Motion Analysis
	Dynamic reactive				(Crossover study)		Spring-loaded tripping board
							(perturbed walking)
Van Wouwe et al. [38]	Static reactive	Yes	No/Not mentioned	No	Control intervention	No	The CAREN system (perturbed standing, beam walking test, perturbed walking)
	Dynamic						
	Dynamic reactive						
Yuzlu et al. [39]	Static	No	No/Not mentioned	No	Control intervention	No	BBS
	Static proactive						TUG
	Static reactive						TUG cognitive
	Dynamic						10MWT
	Dynamic proactive						10MWT dual task
Alizadehsaravi et al. [40]	Dynamic	Yes	No/Not mentioned	No	None	No	3D Motion Analysis (virtual beam walking and normal walking)
							Standing on robot-controlled platform
Alizadehsaravi et al. [41]	Static	Yes	No	Yes	None	No	3D Motion Analysis (perturbed stance on robot-controlled balance platform)
	Static reactive						
Bao et al. [42]	Static	No	No/Not mentioned	No	Control intervention	No	TUG cognitive
	Static proactive						TUG
	Dynamic						FRT
							5STS
							4SST
							Dynamic posturography (SOT)
	Dynamic proactive						Mini BESTest 28
							Mini BESTest 32
Mahjur & Norasteh [43]	NA	No	No/Not mentioned	Yes	Control intervention	No^#^	BESTest
							TUG
Park [44]	Dynamic	No	No/Not mentioned	No	Control intervention	No^#^	OLS
	Dynamic proactive						TUG
Gerards et al. [16]	Static reactive	No	NA	No	Control intervention	No^#^	Mini BESTest
			(voluntary physiotherapy)				
	Dynamic reactive						
	Dynamic proactive						
Lee, Chun & Lee [45]	Static	No	No	Yes	Control intervention	No	BBS
	Dynamic						OLS—EO/EC
	Dynamic proactive						TUG
Lugade et al. [46]	Static	No	No/Not mentioned	No	None	No^#^	TUG
	Static proactive						Dynamic posturography (SOT)
	Static reactive						3D Motion Analysis
	Dynamic						
	Dynamic reactive						
	Dynamic proactive						

***Note*:** TUG–Timed Up and Go test; BBS–Berg Balance Scale; POMA–Tinetti Performance Oriented Mobility Assessment; 5STS–Five Times Sit to Stand test; 4SST–Four Square Step test; OLS–One leg stance; MSL–Maximum Step Length test; FRT–Functional Reach Test; SOT–Sensory Organization Test; FAB scale–Fullerton Advanced Balance Scale; 10MWT– 10 Meter Walk Test; BESTest–Balance Evaluation Systems Test; EO–eyes open; EC–eyes closed; BT–balance training; VT–virtual reality; APA–additional physical activity.

^#^ Training adherence (number of training sessions attended and completed) for experimental group/groups was listed.

*Only 1 or 2 training units were evaluated.

^¥^ Based on the description of the used training exercises, most often games, it was not possible to determine the type of balance.

According to Shumway-Cook, & Woollacott [15], Gerards et al. [16], Zhao et al. [47], we categorized balance into: ***Static*—**the ability to maintain a stable position while sitting or standing; ***Dynamic****—*the ability to maintain balance during movement or when changing from one balanced position to another; ***Static proactive****—*the ability to control posture in response to an anticipated balance disturbance while sitting or standing; ***Dynamic proactive****—*the ability to control posture in response to an anticipated balance disturbance during movement or when changing from one balanced position to another; ***Static reactive****—*the ability to maintain balance after externally induced disturbance to body while standing or sitting; ***Dynamic reactive****—*the ability to maintain balance after externally induced disturbance to body while moving or changing from one balanced position to another.

## Discussion

We found a greater number of women participating in the balance training studies than men (462 vs 300). One of the explanations could be that women in general are more physically active than men [48]. Moreover, they are more willing to participate in the research and undergo physical interventions [49].

Another finding is that studies varied widely in the length of the intervention, number of training sessions and their duration. A dose-response analysis of Gebel et al. [50] revealed that the frequency and duration of balance training are not the modalities that would strongly affect postural balance control in young healthy individuals. Whether this also applies to healthy older adults is unknown. In this context, it is also difficult to draw any comprehensive conclusions regarding the effectiveness of the intervention on balance. Namely, statistically significant improvement did not always occur in all included balance tests. Since the tests used did not always reflect the type of balance being trained, any attempt to draw conclusions about effectiveness would be considerably complicated.

### Entry criteria

We have identified two significant shortcomings of the studies related to the entry criteria. The first one concerned missing a priori criteria for training session attendance. Only 2 out of 26 studies specified requirements for the minimum number of completed training units in their protocols [23, 35]. Six studies reported training adherence [16, 28, 30, 43, 44, 46]. It ranged from 75 to 100%, depending on the study. Low attendance rate could affect exercise dose and consequent training outcomes. According to Williams et al. [21], if training session attendance rates and study outcomes are reported, it should be possible to calculate dose-response characteristics and determine whether an optimal criterion level for training attendance exists. Explicit reporting requirements for the minimum number of completed training units within protocols could help in decision about the level and value of a priori criteria for training session attendance [21].

According to Huang & Yamamoto [51], the weekly training frequency is an important modality of balance control in healthy adults. Authors DiStefano, Clark & Padua [52] found that subjects with more training sessions achieved greater improvements in balance than the subjects who attended less sessions.

The second shortcoming is the missing criterion for additional physical activity in most studies. Only five studies prohibited or restricted the performance of any physical activity during the duration of the intervention [23, 31, 32, 41, 45] and three studies excluded physical activities that explicitly included balance components within the entry criteria [41, 43, 44]. On the contrary, we have identified 1 study where older adults performed physical therapy along with balance training [16]. Therapy was on the voluntary basis and included strength exercises, mobility exercises and/or balance exercises.

According to Taube et al., [53], the neuromuscular adaptation is unique to training level and experience. If we consider that training level and experience are related to the frequency of training units and the number of training stimuli, then additional physical activities that include balance components performed along with the balance intervention could have an impact on training outcome. Physical activity or therapy in general can complicate the response to a balance intervention or conversely, it can interact with the intervention. From the outcome perspective, the intervention can be uniquely effective, but also ineffective [22]. It has been previously observed that even ordinary walking (aerobic activity) significantly improves static and dynamic balance in older adults [54]. Therefore, each physical activity performed along with the intervention should be strictly monitored. Otherwise, it can lead to difficulty in drawing conclusions about the potential utility of the intervention.

### Control group

Five studies did not use a control group [26, 36, 46, 40, 41], but only 3 of them considered this a limitation [36, 40, 41]. A control group is important in increasing confidence in causal inferences by reducing the plausibility of alternative explanations [55, 56].

Three studies used a passive control group that did not receive any intervention or was not allowed to perform or start any physical activity during the study [28, 30, 35]. Using of no-intervention or passive controls in the training studies is increasingly discussed. According to Malay & Chung [57], control group should be free of exposure/intervention under study. On the other hand, there are also opinions that it is not ethical to deny participants an intervention that could benefit them. Louro et al. [58] points out that the use of a no-intervention control group is ethically acceptable if the intervention has not yet been shown to be more beneficial than the no-intervention alternative. However, the possibility that the intervention would be offered to the control group after the end of the study is less discussed. Problem may arise when physical activity is restricted for long time, especially in apparently healthy and physically active older adults. Restriction could lead to a wide array of deleterious social, emotional, and physical changes that can be describe as a downward spiral of health [59]. However, in the case of healthy individuals, temporary restriction of physical activity might not represent a significant problem.

### Training protocol

Detailed description of training protocol should be an integral part of every training study. It means more than providing an intervention length, training frequency and duration. More than half of all studies identified in our review have insufficiently described training protocols [16, 23, 28, 30–32, 34, 36, 39, 42–46]. The most common was missing information about the number of repetitions, sets, and/or rest interval of individual exercises. Description of training exercises and the process of increasing difficulty were also missing. Very few studies specified the type of balance that training was aimed at. Balance training is effective in improving balance tasks that are trained, with limited transfer to non-trained balance tasks [10]. If we were to go into more detail, none of the studies emphasized quality of movement execution. It is one of the biggest keys to great results in all exercise endeavors, especially in the older adults.

A specific balance training studies identified within our review were exergame studies. Some of them lacked game description to be able to identify what type of balance is game aimed at [27, 32, 35, 36]. It was also not stated the duration of the game, number of attempts that were given to participants and rest interval between individual trials/games.

Key training features such as description of individual exercises, number of repetitions and sets, intensity, rest interval, and process of increasing the difficulty can all influence efficacy and reproducibility of the intervention. Even though exercise intensity is the major training stimulus that affects adaptation and overall performance, there is still no methodological approach for its determining in the case of balance exercises [60]. Therefore, we did not consider missing information on exercise intensity as a shortcoming. Indication of exercise dosage is necessary for the sustainability of the training effect and elimination of potential adverse effect of the training intervention [61]. Only by describing the interventions and protocols in detail can other researchers replicate the research or build on the research findings.

### Tests

We have investigated what type of balance the intervention was aimed at, and what diagnostic tools were used to evaluate it. We wanted to know whether the same type of balance that was trained was evaluated. It is known, balance training is effective in improving balance tasks that are trained, with limited transfer on non-trained balance tasks [10]. Therefore, the use of tests that do not reflect the trained type of balance reduces the relevance of the results.

Not all studies used tests that accounted trained type of balance [16, 28, 30, 31, 39]. In four studies, it was not possible to determine the appropriateness of the tests used, due to the lack of training exercises description [32, 35, 36, 43]. One study assessed narrow base walking, while training included proactive and reactive static balance exercises [41]. However, the aim of the study was to evaluate the training transfer from one type of balance to another. In the study of Allin et al. [24], training was aimed to improve reactive balance following perturbations such as slipping and tripping. Body kinematics during perturbed walk, POMA, TUG and OLS were used to evaluate balance control. Three out of four tests used in the mentioned study did not reflect the trained ability. TUG is considered as a test to measure proactive, not reactive balance control [62, 63]. Single leg stance test is used as a test to measure static balance [15, 64]. POMA include tasks to measure static balance and gait [65]. None of the tests involved a reactive component. The goal of the study was not to evaluate the transfer from one type of balance to another. Rezaei et al. [34] used standing on rotating platform as a test to measure dynamic balance. On the contrary, Gerards et al. [66] consider standing on the platform inducing multidirectional shifts or tilts as a test of static reactive balance.

The selection of tests should consider also the physical and functional status of the population involved. Not all tests are suitable for physically active older adults. For instance, OLS has pronounced ceiling effect and, therefore, should not be used as measures of physical performance in high-functioning older adults [67]. However, the physical activity level of older adults was not described in any of the included studies, so it is not possible to determine the appropriateness of the tests used.

The identified shortcomings of the included studies and suggestions for future research are summarized in Table 3.

**Table 3:** Identified gaps and suggestions for future research.

Identified shortcomings	Suggestions for future studies
Missing a priori criteria for session attendance	• Establish minimum criteria within the protocol.
Missing control group	• Include a control group. It is an important aspect of experimental studies that significantly strengthen the findings. • In the case of an active control group, sufficiently describe intervention. This also applies for additional physical activities (APA).
Insufficient or missing information about APA (experimental group)	• Define restrictions on physical activity performed outside of the intervention. • Describe the activity/activities in detail.
Insufficiently described training protocol	• Describe all exercises and training characteristics in detail (number of exercises, order, repetitions, sets, rest interval, time, and increasing difficulty).
Inappropriate selection of tests	• Choose tests in accordance with the aim of the study and those that most reflect the type of balance being trained.

## Limitations

Our study has several limitations that should be addressed. First, the design of the studies within the inclusion criteria was not specified. Although scoping review allows to include a wide range of study designs [8], it would be appropriate to specify them in the case of future research. Second, review studies were excluded to avoid duplication of literature. On the other hand, this type of study design can serve as a source for identifying potentially relevant studies. Third, methodological quality of individual studies was not examined. Fourth, some exergame interventions could be multicomponent. Since the games were often not (well) described, we cannot rule it out. Fifth, important works may have been omitted due to the inclusion of only papers available on open access publication.

## Conclusions

Insufficiently described protocol and inappropriately chosen tests to be the most serious shortcomings of identified studies. For this reason, even with an excellently conducted experiment, it is almost impossible for practitioners to apply the results of such studies into practice. Therefore, researchers should pay more attention to possible users of the acquired knowledge, which is more than desirable in the case of exercise programs for older adults.

## Supporting information

S1 File Protocol [15–22]. (DOCX)

S1 Checklist Preferred Reporting Items for Systematic reviews and Meta-Analyses extension for Scoping Reviews (PRISMA-ScR) checklist. (PDF)
