# Supplementary material for: What are the hidden shortcomings of balance training research in older adults that prevent its transfer into practice? Scoping review
Source: PLoS One. 2025 Jan 2;20(1):e0308752. doi: 10.1371/journal.pone.0308752 (PMC11695024; doi:10.1371/journal.pone.0308752)
Supplement: S1 File — (DOCX) [file pone.0308752.s001.docx]

| ***What type of balance was evaluated?*** (based on the tests used) ^*^   - static - static reactive - static proactive - dynamic - dynamic reactive - dynamic proactive |
| --- |
| *Justification*  Balance control is a skill that is highly specific to a given task. It is therefore necessary to consider several aspects, based on which balance can be categorized as static, dynamic, proactive, and reactive [15]. Authors Gerards et al. [16] in their study even differentiate static reactive balance and dynamic reactive balance. Different types of exercises are intended to produce different outcomes for specific populations [17]. |
| ***Was the intervention sufficiently described?***   - length, training frequency and time (Yes/No) - used exercises (description, order, number of repetitions/sets, rest interval) (Yes/No) - increasing difficulty (Yes/No) - exergames (description, number of repetitions/length, rest interval) (Yes/No) - perturbations (type, direction, repetitions, speed) (Yes/No) |
| *Justification*  Incomplete training intervention description limit exact replication of training units and prevent critical appraisal of interventions when contradictory results are reported. Plowman & Smith [18] consider exercise specificity, frequency, intensity, duration, and rest interval as an important element in designing exercise interventions. |
| ***What type of balance was evaluated?*** (based on the tests used) ^*^   - static - static reactive - static proactive - dynamic - dynamic reactive - dynamic proactive |
| *Justification*  As mentioned above, balance is a skill that is highly specific to a given task. Authors Owings et al. [19] and Mackey & Robinovich [20] found that measures of static stance do not reflect recovery after postural disturbances in older adults. In other words, specific type of balance that was trained should be evaluated. |
| ***Was the minimum required training participation set for the experimental group?*** (Yes/No) |
| *Justification*  Training attendance could impact training load (exercise dose) and consequent training outcome. If training studies report training description, training sessions attendance rates and training outcome, it should be possible to calculate dose–response characteristics and identify whether an optimal criterion level for training session attendance exists [21]. |
| ***Was the experimental group prohibited/restricted from performing any physical activities along with intervention?***   - Yes - No/Not mentioned. It was not performed - No/Not mentioned. It was performed **»** ***Was the activity adequately described?*** (Yes/No)   ***What type of control group was used in the study?***   - none - passive **»** ***Were the participants restricted/prohibited from performing any leisure-time physical activity during the study?*** - Yes - No/Not mentioned. It was not performed - No/Not mentioned. It was performed **»** ***Was the activity adequately described?*** (Yes/No) - control intervention **»** ***Was the training adequately described?*** (Yes/No)   **» *Did the group perform any type of physical activity/therapy along with intervention?***   - No - Yes ***» Was the training/therapy adequately described?*** (Yes/No) |
| *Justification*  The inability to differentiate intervention effects and the effect of additional physical activities can potentially lead to the inability for scientists to ascertain the intervention effectiveness [22]. |
